# Research highlights, *The Plant Genome*, Volume 13, Issue 1

**DOI:** 10.1002/tpg2.20025

**Published:** 2020-05-12

## 1 HIDDEN SOYBEAN HETEROGENEITY

Soybean is a globally important crop with a narrow genetic diversity limiting its improvement. Soybeans are self‐pollinating, so seeds of accessions are typically maintained as pure‐lines and assumed to be virtually genetically identical among individuals. Mihelich et al. (https://doi.org/10.1002/tpg2.20000) discovered within‐line heterogeneity in 4% of 20,087 accessions of the USDA Soybean Germplasm Collection. These detected heterogenous regions collectively span almost the entire soybean genome. This discovery is important for users of crop germplasm seed banks that are otherwise expecting genetic uniformity among individuals of an accession. Furthermore, heterogeneous intervals in genomic regions of interest can be used to identify readily available near isogenic lines for rapid trait mapping, gene validation, and potentially cultivar development.

## 2 IMPROVED GENOME ASSEMBLY OF *Oryza longistaminata*

African wild rice, *Oryza longistaminata*, possesses highly prized traits, such as rhizomatousness for perennial rice breeding, tolerance to stresses, and high biomass production on poor soils. A high‐quality genome assembly is fundamental to mine novel alleles that are of great significance in breeding new rice cultivars. Li et al. (https://doi.org/10.1002/tpg2.20001) report and annotate an improved genome assembly of *O. longistaminata*. Comparative genomic analysis reveals that lineage‐specific expansion of gene families relevant to biotic and abiotic stresses. This dataset will enlarge the existing database of rice genome resources and accelerate the exploration of novel genes from wild rice to enhance rice breeding programs.

## 3 GENOMIC SELECTION IS PROMISING FOR LENTIL BREEDING

Genomic selection is a marker‐based selection strategy suggested to improve the genetic gain of quantitative traits but it has not been tested for use in lentil (*Lens culinaris* Medik.) breeding. Haile et al. (https://doi.org/10.1002/tpg2.20002) showed that using multiple‐trait prediction models and models that account for genotype × environment interaction improve accuracy by 14 and 66%, respectively, for a low heritability trait. When trait heritability is high and no large effect quantitative trait loci underlie traits, however, most genomic prediction models have similar accuracy. Genomic selection can be implemented in lentil breeding to make predictions within populations and across environments, but across‐population prediction should not be considered when the population size is small.

## 4 ROOT ANATOMICAL TRAITS ARE PLASTIC

Root anatomical phenes have important roles in soil resource capture and plant performance; however, their phenotypic plasticity and genetic architecture is poorly understood. Schneider et al. (https://doi.org/10.1002/tpg2.20003) phenotyped root anatomy in a large maize association panel in the field with and without water deficit stress. Anatomical phenes displayed stress and environmental plasticity; many phenotypic responses to water deficit were adaptive, and the magnitude of response varied by genotype. We identified candidate genes associated with stress and environmental plasticity and expression of phenes under well‐watered and water‐stress conditions. The genetic architecture of phenotypic plasticity is highly quantitative, and many distinct genes control plasticity in response to water deficit and different environments, which poses a challenge for breeding programs.

## 5 BREEDING BETTER OATS

Oats are an important cereal grain for human nutrition and animal feed but progress in the use of genomic‐based breeding methods has been restricted by complexity and cost. Mellers et al. (https://doi.org/10.1002/tpg2.20004) report the successful implementation of genomic prediction using the stratified progeny of a cross between two cultivated winter oat varieties. This allowed for genomic prediction at both early and later generations, supporting earlier selection for multiple target environments within a breeding program. The study also demonstrates that mixed genetic data types can be integrated allowing the use of legacy data. Taken together, the findings demonstrate how small breeding programs can test and initiate genomic predictions building on existing data and resources.

## 6 CHOOSING OPTIMAL POPULATION FOR GWAS

How to choose a suitable population for a genome‐wide association study (GWAS) has not been discussed extensively so far. The tradeoff between sample size and the effect of population structure should be discussed more. Hamazaki et al. (https://doi.org/10.1002/tpg2.20005) reported that mixed populations with different genetic backgrounds could improve the detection power of causal variants. Simulation studies were conducted for seven unmixed and mixed populations, and the detection power of GWAS was compared across the seven populations. As a result, the target population with a narrow genetic background plus a diverse population improved the detection power of causal variants. Thus, germplasm collections with public sequence data are suggested as being useful for improving the detection power of GWAS.

## 7 IDENTIFY MARKERS ASSOCIATED WITH YIELD TRAITS OF SUGARCANE

To meet the increased demand for sugar and biofuel supplies, it is critical to breed sugarcane cultivars with robust performance in yield traits. Markers associated with the yield traits of sugarcane are important genetic tools for crop improvement. Yang et al. (https://doi.org/10.1002/tpg2.20006) analyzed the association of nine yield traits, including stalk number, stalk diameter, internode length, leaf width, leaf length, Brix, dry weight, total weight, and water content, in a sugarcane diversity panel consisting of 308 accessions with 74,900 DNA markers. A total of 217 DNA markers significantly associated with yield traits were identified through a genome‐wide association study with models tailored for polyploid species. The results of this study not only provided the genetic bases of multiple yield traits in sugarcane, but also suggested important tools and deposited valuable genomics resources for the sugarcane community. The new methods and concepts in this study shed light on research in sugarcane and polyploid species.

## 8 DETECTED RESISTANCE LOCI CHANGE OVER SEASON

Plant diseases are the result of complex interactions among plants, pathogens, and the environment. To date, most investigations of disease resistance measure disease severity at a single time point within each environment, which ignores the possible impact of temporal variation. McNish et al. (https://doi.org/10.1002/tpg2.20007) discovered that the genetic loci associated with oat crown rust resistance change over time within an environment. Few loci were detected throughout a season and many loci were detected briefly. Thus, experiments that consider the temporal variation of disease development may more fully characterize the genetic architecture of resistance for dynamic plant diseases.

## 9 ELEVATING CAROTENOIDS IN SWEET CORN

Sweet corn is a highly consumed vegetable in the United States, but its contribution to the daily intake of carotenoids (provitamin A carotenoids, lutein, and zeaxanthin) important for human health and nutrition is limited. Baseggio et al. (https://doi.org/10.1002/tpg2.20008) conducted a genome‐wide association study of fresh kernel carotenoid variation in a panel of sweet corn inbred lines. They found an association of *β‐carotene hydroxylase* (*crtRB1*) with the concentration of β‐carotene (a provitamin A carotenoid) and *lycopene epsilon cyclase (lcyE*) with the ratio of flux between the α‐ and β‐carotene branches in the carotenoid biosynthetic pathway. The most favorable *lcyE* allele and *crtRB1* haplotype for increasing β‐branch carotenoids (β‐carotene and zeaxanthin) and β‐carotene, respectively, were found to be uncommon (≤5%) for sweet corn lines possessing the *shrunken2* mutation, thus these uncommon variants are important targets of selection. Whole‐genome prediction of carotenoids, especially lutein, resulted in moderately high predictive abilities, suggesting that these phenotypes are amenable to genomic selection in sweet corn breeding programs.

## 10 SORGHUM HAPLOTYPE DATABASE IMPROVES IMPUTATION

Accelerating cultivar development requires plant breeders to manage and use large genomic datasets while still minimizing the cost and quantity of sequence data that needs to be generated for each cycle of selection. Jensen et al. (https://doi.org/10.1002/tpg2.20009) built a haplotype database in sorghum that combines existing whole genome sequence information from a diverse set of individuals into a single pan‐genome. The database structure, called the Practical Haplotype Graph, organizes genome sequence into conserved haplotype blocks while maintaining variant information from reference individuals. Even when using extremely low‐coverage or random sequence data, the sorghum Practical Haplotype Graph can accurately impute genotypes for new individuals. This can decrease genotyping costs and merge genotype calls from a variety of genotyping methods, making genomic selection and larger breeding populations more accessible. In this paper, the authors demonstrate that the sorghum Practical Haplotype Graph is a useful research and breeding tool that maintains variant information from a diverse group of taxa, stores whole‐genome sequence data in a condensed but accessible format, unifies genotypes across genotyping platforms, and provides a cost‐effective option for genomic selection.

## 11 TRANSPOSON DRIVEN OLIVE GENOME EVOLUTION

The study of archeological remains date the primary domestication of olive (*Olea europaea* L.) in the Neolithic period, around 6,000–5,500 BC. Most of the cultivated olive trees are reproduced clonally, with a reduced number of sexual crosses, so the molecular mechanisms, such as recombination, may have a limited influence in the development of new varieties. Jiménez‐Ruiz et al. (https://doi.org/10.1002/tpg2.20010) investigates possible sources of genetic variation during the olive domestication process. The olive genome of the popular ‘Picual’ cultivar was sequenced and assembled, setting up a new reference genome for this important crop. Then, the resequencing of a panel of 40 cultivated and 10 wild accessions was used to elucidate the evolution of the olive genome during the domestication process. Population analyses support two domestication events for olive trees, including an early possible genetic bottleneck. Despite genetic bottlenecks, cultivated accessions showed a high genetic diversity driven by the activation of transposable elements. Several families of transposable elements were expanded in the last 5,000 or 6,000 years and produced insertions near genes that may have been involved in selected traits during domestication such as reproduction, photosynthesis, seed development, and oil production. Therefore, a great genetic variability has been found in cultivated olive as a result of a significant activation of transposable elements during the domestication process.

## 12 VARIANCE HETEROGENEITY ANALYSIS IDENTIFIES EPISTASIS

Variance heterogeneity quantitative trait loci (vQTL) affect the variability of traits, which cannot be detected through standard GWAS (genome‐wide association studies). Hussain et al. (https://doi.org/10.1002/tpg2.20011) used variance heterogeneity GWAS to investigate loci that control the variability of grain cadmium concentration in bread wheat. Novel vQTL on chromosomes 2A and 2B and mvQTL (QTL affecting both mean and variability) on chromosome 5A were identified. The presence of variance heterogeneity was explained by epistatic interactions between vQTL and mvQTL. Homoeology and interactions within the vQTL on chromosomes 2A and 2B were also found. Collectively, this study shows that epistasis is an essential component of genetic variation for genetic regulation of grain cadmium concentration in wheat.

Intermediate wheatgrass is a dual‐use perennial crop with nutritious grain and several environmental benefits. Currently at an early stage of domestication, several key traits need improvement for its successful mainstream adoption. Genomic selection has been a key tool in expediting the domestication timeline of the crop. Bajgain et al. (https://doi.org/10.1002/tpg2.20012) report that using GxE effects in genomic prediction models significantly boost trait predictions in a large, non‐phenotyped breeding population. Incorporating trait data from correlated environments greatly improved predictions for multiple traits such as grain yield, spike weight, and free grain threshing. Implementing models that utilize GxE information can further help in improving agronomic and domestication traits in intermediate wheatgrass.

## 13 SORGHUM GENES MODIFY CAROTENOID CONCENTRATIONS

Vitamin A deficiency is one of the most prevalent nutritional deficiencies worldwide. Sorghum is a staple cereal that could help decrease vitamin A deficiency if its grain carotenoid concentrations were higher. Cruet‐Burgos et al. (https://doi.org/10.1002/tpg2.20013) report that variation in sorghum grain carotenoid concentrations is controlled by a small number of genes. Lutein, zeaxanthin, and β‐carotene were the three carotenoid compounds present in measurable amounts in the sorghum grain. Genetics played a larger role in carotenoid concentration than environment, although environment also affected concentrations. A genome‐wide association study showed that around 12 genes are involved in carotenoid variation, particularly *zeaxanthin epoxidase* (*ZEP*). The genes found in this study could be used in breeding to increase carotenoid concentrations in sorghum grain.

## 14 IMPROVING INDUCERS WITH GENOMIC PREDICTION

Maternal haploid inducers are used in the production of maize doubled haploid (DH) lines. Other studies have reported that their induction ability, commonly referred to as haploid induction rate (HIR), is under polygenic control. Since HIR is a very time consuming and laborious trait to phenotype, genomic prediction (GP) could be a cost‐efficient method to improve inducers. In this study, Almeida et al. (https://doi.org/10.1002/tpg2.20014) observed that GP can be successfully applied for the improvement of HIR and of different agronomic traits of importance to inducers. They also found that GP can be applied for parental selection and for predicting the performance of new inducer breeding populations. Improving inducer performance is important because it can significantly decrease the cost of DH line production.

